# Identification and Comparative Analysis of Venom Proteins in a Pupal Ectoparasitoid, *Pachycrepoideus vindemmiae*

**DOI:** 10.3389/fphys.2020.00009

**Published:** 2020-01-24

**Authors:** Lei Yang, Yi Yang, Ming-Ming Liu, Zhi-Chao Yan, Li-Ming Qiu, Qi Fang, Fang Wang, John H. Werren, Gong-Yin Ye

**Affiliations:** ^1^State Key Laboratory of Rice Biology, Ministry of Agriculture and Rural Affairs Key Lab of Molecular Biology of Crop Pathogens and Insects, Institute of Insect Sciences, Zhejiang University, Hangzhou, China; ^2^Department of Biology, University of Rochester, Rochester, NY, United States

**Keywords:** parasitoid, venom, *Pachycrepoideus vindemmiae*, *Drosophila*, Pteromalidae

## Abstract

Parasitoid wasps inject venom containing complex bioactive compounds to regulate the immune response and development of host arthropods and sometime paralyze host arthropods. Although extensive studies have been conducted on the identification of venom proteins in larval parasitoids, relatively few studies have examined the pupal parasitoids. In our current study, a combination of transcriptomic and proteomic methods was used to identify 64 putative venom proteins from *Pachycrepoideus vindemmiae*, an ectoparasitoid of *Drosophila*. Expression analysis revealed that 20 tested venom proteins have 419-fold higher mean expression in the venom apparatus than in other wasp tissues, indicating their specialization to venom. Comparisons of venom proteins from *P. vindemmiae* and other five species spanning three parasitoid families detected a core set of “ancient” orthologs in Pteromalidae. Thirty-five venom proteins of *P. vindemmiae* were assigned to the orthologous groups by reciprocal best matches with venoms of other pteromalids, while the remaining 29 were not. Of the 35 categories, twenty-seven have orthologous relationships with *Nasonia vitripennis* venom proteins and 25 with venoms of *Pteromalus puparum*. More distant relationships detected that five and two venom proteins of *P. vindemmiae* are orthologous with venoms of two Figitidae parasitoids and a Braconidae representative, respectively. Moreover, twenty-two venoms unique to *P. vindemmiae* were also detected, indicating considerable interspecific variation of venom proteins in parasitoids. Phylogenetic reconstruction based on a set of single-copy genes clustered *P. vindemmiae* with *P. puparum*, *N. vitripennis*, and other members of the family Pteromalidae. These findings provide strong evidence that *P. vindemmiae* venom proteins are well positioned for future functional and evolutionary studies.

## Introduction

Hymenopteran parasitoids dampen oscillations of insect populations, including those of agricultural and livestock pests. Parasitoids differ in egg-laying behavior (Pennacchio and Strand, 2006). Ectoparasitoids lay eggs on the surface of the host. In contrast, endoparasitoids oviposit into the host hemocoel (Asgari and Rivers, 2011). To assist successful development of offspring, female wasps inject multiple virulence factors into the host, including venom proteins (Asgari and Rivers, 2011; Moreau and Asgari, 2015; Wan et al., 2019), polydnaviruses (PDVs) (Ye et al., 2018), ovarian fluids (Teng et al., 2019), virus-like particles (VLPs) (Harvey et al., 2013). Venom proteins, an indispensable part of virulence factors, inhibit immunity, interrupt development, and regulate metabolism of the host (Asgari and Rivers, 2011; Moreau and Asgari, 2015).

Venom proteins comprise various bioactive molecules, such as enzymes, protease inhibitors, recognition and binding proteins, and other unknown compounds (Asgari and Rivers, 2011; Moreau and Asgari, 2015). So far, many venom components have been extensively investigated, such as RhoGAP (Labrosse et al., 2005a, b; Colinet et al., 2007), calreticulin (CRT) (Zhang et al., 2006; Wang et al., 2013; Siebert et al., 2015), sarco/endoplasmic reticulum calcium ATPase (SERCA) (Mortimer et al., 2013), GH1 β-glucosidase (Hubert et al., 2016), α-amylases (Wang et al., 2019), Crp32B (Teng et al., 2019). They inhibit host cell spreading, prevent host cellular encapsulation, suppress host hemolymph melanization or hydrolyze nutrients of the host to guarantee survival of wasp progeny. Besides, venom proteins also cooperate with other virulence factors to outwit the host. For instance, venom proteins of endoparasitoids, *Cotesia melanoscela*, *Pseudoplusia includens* and *Cotesia rubecula*, acted synergistically with PDVs in altering host physiological status (Stoltz et al., 1988; Michael and Noda, 1991; Zhang et al., 2004b). Additionally, previous studies have demonstrated the synergized effects of venom and calyx fluids on host’s development and immune responses (Strand and Dover, 1991; Teng et al., 2016).

Identification of these venom proteins is the first step to study their functions. Recently, high-throughput sequencing and mass spectrometry have made it technically feasible to isolate and identify venom proteins in parasitoids (Danneels et al., 2010; Vincent et al., 2010; Goecks et al., 2013; Mortimer et al., 2013; Hubert et al., 2016; Laurino et al., 2016; Yan et al., 2016; Teng et al., 2017), as well as in snakes (Ching et al., 2006), scorpions (Ma et al., 2010), and spiders (Haney et al., 2014). Although there are as many as 600,000 parasitoid species (Heraty, 2009), few exhaustive studies have been carried out on their venom compositions and functions. To date, venom proteins of endoparasitoids are relatively well studied (Labrosse et al., 2005a, b; Colinet et al., 2009; Furihata et al., 2013; Goecks et al., 2013; Heavner et al., 2013; Mortimer et al., 2013). However, the venom protein repertoire of ectoparasitoids is less well known (Marchiori and Barbaresco, 2007; Marchiori et al., 2013; Chen et al., 2015; Marchiori and Borges, 2017).

*Pachycrepoideus vindemmiae* belongs to the family Pteromalidae (Hymenoptera). It is a versatile and solitary pupal ectoparasitoid that parasitizes various flies, including the genera of *Drosophila*, *Musca*, *Anastrepha*, *Calliphora*, and so on (Marchiori and Barbaresco, 2007; Marchiori et al., 2013; Marchiori and Borges, 2017). In this study, we examined the diversity of *P. vindemmiae* venom compositions based on both transcriptome-sequencing and proteome analysis, and compared it to venoms of three parasitoid families. Given the amazing toolkit available in its host drosophilids, we propose that future studies combining the power of *Drosophila* as a model system with its ectoparasitoid *P. vindemmiae* have great potentials for advancing our understanding of the functions and evolution of venom proteins, and assessing their pharmacological possibilities (Danneels et al., 2010, 2015; Moreau and Asgari, 2015; Huerta-Rey et al., 2017).

## Materials and Methods

### Insect Rearing

The *Pachycrepoideus vindemmiae* colony was kindly provided by Prof. Yongyue Lu (South China Agricultural University, Guangzhou, China) in January 2016. Subsequently, *P. vindemmiae* was maintained with *D. melanogaster* pupae at 25°C, with a photoperiod of 14:10 hr (light:dark), as described (Chen et al., 2015). After eclosion, the adults were held in glass containers and fed with 10% (v/v) honey solution.

### Venom Apparatus Collection and Isolation of Total RNA

Mated female wasps aged 2–5 days were anesthetized at 4°C for 10 min, rinsed in 75% ethanol (v/v) once, and then rinsed in sterile phosphate-buffered saline (PBS, pH 7.2) thrice. Subsequently, the females were dissected in PBS containing 1 unit/μL Murine RNase inhibitor (Vazyme, Nanjing, China) on an ice plate under a Leica MZ 16A stereomicroscope (Leica, Wetzlar, Germany), the venom apparatus (venom reservoirs and associated glands, henceforth, called the VG) and carcasses (the female body minus venom apparatus, henceforth, called the CA) were collected into 1 mL TRIzol reagent (Invitrogen, Carlsbad, CA, United States), respectively. Total RNA was extracted according to the manufacturer’s protocol. RNA degradation and contamination were monitored on 1% agarose gels. RNA purity was checked using the NanoPhotometer^®^ spectrophotometer (IMPLEN, CA, United States). RNA concentration was measured using the Qubit^®^ RNA Assay Kit in Qubit^®^ 2.0 Flurometer (Life Technologies, CA, United States). RNA integrity was assessed using the RNA Nano 6000 Assay Kit of the Agilent Bioanalyzer 2100 system (Agilent Technologies, CA, United States).

### Construction and Sequencing of the cDNA Library

A total amount of 1.5 μg RNA per sample was used as input material for the RNA sample preparations. Sequencing libraries were generated using the NEBNext^®^ Ultra^TM^ RNA Library Prep Kit for Illumina^®^ (NEB, United States) following the manufacturer’s recommendations and index codes were added to attribute sequences to each sample. Briefly, mRNA was purified from total RNA using poly-T oligo-attached magnetic beads. Fragmentation was performed using divalent cations under elevated temperature in NEBNext First Strand Synthesis Reaction Buffer (5X). First strand cDNA was synthesized using random hexamer primer and M-MuLV Reverse Transcriptas. Second strand cDNA synthesis was subsequently performed. The remaining overhangs were converted into blunt ends via exonuclease/polymerase activities. After adenylation of 3′ ends of DNA fragments, NEBNext adaptors with hairpin loop structure were ligated to prepare for hybridization. To select cDNA fragments of preferentially 150∼200 bp in length, the library fragments were purified with AMPure XP system (Beckman Coulter, Beverly, United States). Then, 3 μl USER Enzyme (NEB, United States) was used with size-selected, adaptor-ligated cDNA at 37°C for 15 min followed by 5 min at 95°C before PCR. PCR was performed with Phusion High-Fidelity DNA polymerase, Universal PCR primers and Index (X) Primers. Eventually, PCR products were purified (AMPure XP system), library quality was assessed on the Agilent Bioanalyzer 2100 system. The clustering of the index-coded samples was performed on a cBot Cluster Generation System using the TruSeq PE Cluster Kit v3-cBot-HS (Illumia) according to the manufacturer’s instructions. After cluster generation, the library preparations were sequenced on an Illumina Hiseq platform and paired-end reads were generated.

### Transcriptomic Data Analysis

Clean data were obtained by processing raw data of fastq format through in-house perl scripts. In this step, reads containing adapters, reads containing ploy-N and low quality reads were removed. At the same time, Q20, Q30, GC-content, and sequence duplication levels of the clean data were calculated. Then, clean reads were assembled using Trinity v2012-10-05 without a reference genome (Grabherr et al., 2011). After assembling, the longest cluster sequences from each transcript were chosen as the reference sequences for subsequent analyses (henceforth, called unigenes). All unigenes were annotated on NCBI non-redundant protein sequences (Nr) database using blastx with *e*-value < 1e^–5^. We estimated the expression levels of transcripts using the software RSEM (Li and Dewey, 2011). Differentially expressed unigenes were defined using DESeq software with strict screening thresholds of a corrected *p*-value < 0.05, | log_2_ (VG readcount/CA readcount)| > 1 and FPKM ≥ 10 (Anders and Huber, 2010). Gene Ontology (GO) enrichment analysis was implemented using the GOseq R packages based on Wallenius non-central hyper-geometric distribution (*e*-value < 1e^–6^) (Young et al., 2010). Additionally, KOBAS software was used for testing the statistical enrichment of differential expression genes in Kyoto Encyclopedia of Genes and Genomes pathways (KEGG, *e*-value < 1e^–10^) (Mao et al., 2005).

### Venom Protein Collection

Mated female wasps aged 2–5 days were anesthetized at 4°C for 10 min as described above, and then dissected in sterile PBS containing 1 mM ProteinSafe^TM^ Protease Inhibitor Cocktail (Transgen, Beijing, China) on an ice plate under a stereoscope (Leica, Wetzlar, Germany). The venom reservoir was separated and washed thrice, and then transferred into 1.5 mL Eppendorf tubes. After centrifugation at 18,000 *g* for 10 min, the supernatant was transferred into a new 1.5 mL Eppendorf tube and stored at −80°C until use. The concentration of the venom proteins was determined by a Modified Bradford Protein Assay Kit (Sangon Biotech, Shanghai, China) according to the manufacturer’s protocol.

### SDS-PAGE and LC-MS/MS Analyses of Venom Proteins

Proteins from *P. vindemmiae* venom reservoirs were separated by 12% sodium dodecyl sulfate polyacrylamide gel electrophoresis (SDS-PAGE) and stained with Coomassie Brilliant Blue R-250. After quality inspection, the solution containing venom proteins was digested into peptides with trypsin and analyzed on a liquid chromatography tandem mass spectrometry (LC-MS/MS) system (LTQ-VELOS; Thermo Finnigan, San Jose, CA, United States). Subsequently, samples were desalted on Zorbax 300 SB-C18 columns (Agilent Technologies, Wilmington, DE, United States), and then separated on a RP-C18 column (150 m i.d., 150 mm length) (Column technology Inc., Fremont, CA, United States). Buffer A was water with 0.1% formic acid; Buffer B was 84% acetonitrile with 0.1% formic acid. The Buffer B gradient was: 0–3 min, from 3% to 9%; 3–93 min, from 9% to 32%; 93–108 min, from 32% to 40%; 108–113 min, from 40% to 100%; and 113–120 min, 100%. The raw data from one proteome were generated and the identified peptide fragments were searched against the translated transcriptomic sequences of VG using the Sequest search algorithm (Eng et al., 1994). The parameters were set as follows: carbamidomethyl was set as a fixed modification, and oxidation was set as a variable modification, the cross-correlation scores (Charge = 1, XCorr ≥ 1.9; Charge = 2, XCorr ≥ 2.2; Charge = 3, XCorr ≥ 3.75, and delta CN ≥ 0.1) were used as the filter criteria. This part of experiment was conducted by Shanghai Applied Protein Technology Co., Ltd. (Shanghai, China).

### qPCR

Total RNA of the VG and CA were separately extracted. cDNA was synthesized from 1 μg RNA using the TransScript One-Step gDNA Removal and cDNA Synthesis SuperMix (Transgen, Beijing, China). All specific primers for qPCR were designed by AlleleID 6 software (PREMIER Biosoft, Palo Alto, CA, United States). The qPCR was run in the CFX96^TM^ Real-Time PCR Detection System (Bio-Rad, Hercules, CA, United States) using ChamQ SYBR qPCR Master Mix (Vazyme, Nanjing, China) according to the manufacturer’s protocol. The programs were set as follows: enzyme activation at 95°C for 30 s, followed by 40 cycles with denaturation at 95°C for 5 s, annealing and extension at 60°C for 30 s, and a melting curve analysis. mRNA expression levels were normalized to the reference (28S rRNA) (Ballinger and Perlman, 2017), and quantified based on the comparative 2^–ΔΔCT^ method (Livak and Schmittgen, 2001). The experiments were repeated 3 times.

### Sequence Analysis and Phylogenetic Construction of Venom Proteins

The online software SignalP 4.1 was used for predicting signal peptides. Protein tertiary structure was modeled by the homology-modeling server SWISS-MODEL, as described (Biasini et al., 2014). Multiple sequence alignment was performed by Clustal Omega, and visualized using the ESPript 3.0 server (Robert and Gouet, 2014). We used a web resource, Simple Modular Architecture Research Tool, for identification and annotation of protein domains (Letunic and Bork, 2017). The motif-based sequence logo was generated using WebLogo (Crooks et al., 2004). For phylogenetic construction of “His_Phos_2 domain,” low quality regions were removed by Gblocks Server. Subsequently, the phylogenetic tree was constructed by Mega 6 software using the maximum likelihood method with 1000 bootstrap values (Tamura et al., 2013), and visualized using the interactive tree of life (iTOL) v3 (Letunic and Bork, 2016).

### Comparative Analysis of Parasitoid Venom Repertoires

Venom protein sequences of six parasitoids were obtained from previous literature (De Graaf et al., 2010; Goecks et al., 2013; Yan et al., 2016; Teng et al., 2017), including 55 entries in *Cotesia chilonis*, 169 in *Leptopilina boulardi*, 176 in *Leptopilina heterotoma*, 79 in *N. vitripennis*, 70 in *P. puparum* and 64 in *P. vindemmiae* (this study). Venom proteins were assigned to orthologous groups using OrthoMCL with a cutoff *p*-value of 1e^–5^ (Doerks et al., 2002). The categories best-reciprocal matches with other parasitoids’ venom proteins were defined as orthologs, and vice versa, as species specific venom proteins. Then, the numbers of orthologs and species specific venom proteins were counted, respectively, and a clustered heatmap was constructed using TBtools v0.6669 based on the proportion of orthologs in total venom proteins (Chen et al., 2018).

### Phylogeny of *Pachycrepoideus vindemmiae*

The phylogeny was reconstructed based on a data set of 107 proteins (Lindsey et al., 2018). Protein sequences from *P. vindemmiae* (this study), *P. puparum* (GECT00000000.1), *Aphelinus abdominalis* (GBTK00000000.1), and *Leptomastix dactylopii* (GBNE00000000.1) transcriptomes were assigned to these 107 protein groups by the best-reciprocal hits from searching between the translated transcriptome and the *N. vitripennis* genome (OGSv2.0^1^). Blastp was conducted with an *e*-value cut-off < 1e^–5^. All 107 protein sequences were aligned by Mafft v7.123b, using the L-INS-i alignment algorithm (Katoh and Standley, 2013). The alignments were filtered by trimAI version 1.2rev59, with automated settings, and then concatenated using AMAS. The tree was conducted using RAxML v8.0.20 by setting the substitution model as “PROTGAMMAAUTO” with 1000 bootstraps (Stamatakis, 2014), and visualized with iTOL v3 (Letunic and Bork, 2016).

## Results and Discussion

### Identification of Venom Proteins by Transcriptomic Method

#### Analyses of Venom Apparatus and Carcasses Transcriptome

Four cDNA libraries were separately generated and then sequenced, including three replicates from *P. vindemmiae* VG and one from CA. We obtained 50,757,700 bp, 50,391,012 bp, 42,563,398 bp, and 47,774,240 bp sequenced raw reads from VG1, VG2, VG3, and CA, respectively. Then, raw reads were filtered to eliminate low quality reads, and 49,355,482 bp clean reads for VG1, 48,780,604 bp for VG2, 41,347,730 bp for VG3, and 46,348,590 bp for CA were acquired. Assembly statistics showed that the transcripts result in N50, N90, and mean lengths of 2,186 bp, 267 bp, and 819 bp. For all transcripts, the longest from the same transcription locus were regarded as unigenes. Finally, 61,747 unigenes representing 161,819 transcripts were obtained. N50, N90, and the mean lengths were 3,566 bp, 622 bp, and 1,684 bp. Among 61,747 unigenes, 36,122 (58.5%) got matches in the Nr database using blastx with an *e*-value < 1e^–5^.

#### Functional Characterization of Upregulated Unigenes in Venom Apparatus

To define a robust set of venom proteins by transcriptomic method, we assumed that venom proteins were significantly higher expression in VG relative to CA. Hereby, differentially expressed genes were defined with the screening thresholds of a corrected *p*-value < 0.05, | log_2_ (VG readcount/CA readcount)| > 1 and FPKM ≥ 10. By these criteria, 398 differentially expressed unigenes were identified, including 335 upregulated unigenes in VG (UVG) compared to CA (Supplementary Table S1) and 63 downregulated items. We annotated the 335 UVG in the GO consortium database. Genes categorized as having “hydrolase activity” (GO: 0016787) were most abundant (97) (Supplementary Figure S1). It is inferred that the “hydrolase activity” proteins participate in immune and metabolic regulation of the host. In contrast, the 63 downregulated unigenes were enriched for GO categories myosin complex, actin cytoskeleton and motor activity.

To represent our knowledge of 335 UVG on molecular interaction, reaction and relation networks, their participation in KEGG pathways were assessed. On the basis of annotations, we sorted them into different categories. Among the top 20 annotated pathways, the most enriched categories pertained to RNA degradation (5 unigenes) and aminoacyl-tRNA biosynthesis pathways (5 unigenes) (Supplementary Figure S2). These unigenes may regulate host metabolism, degrade host nutrients and eventually provide additional energy for wasp larval development.

### Identification of Venom Proteins by Proteomic Approach

**FIGURE 1:**
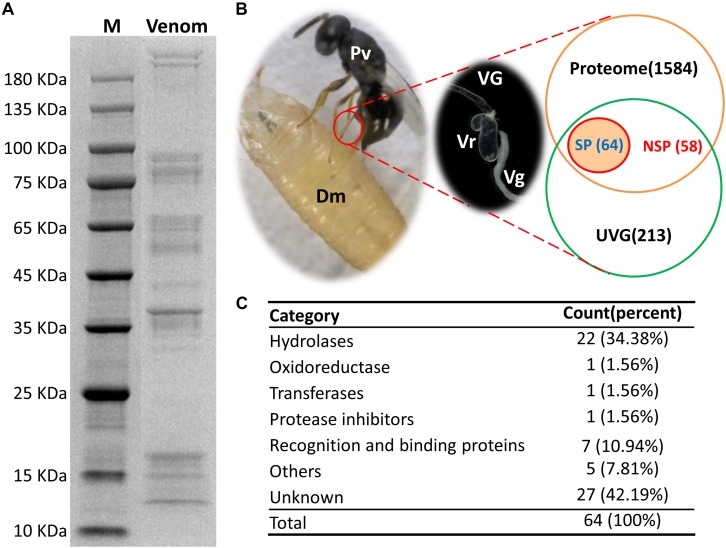
Identification of putative venom proteins in *Pachycrepoideus vindemmiae* combining transcriptomic and proteomic analyses. **(A)** 12% SDS-PAGE analysis of *P. vindemmiae* venom proteins followed by Coomassie Brilliant Blue staining. “M” denotes molecular weight marker. **(B)** Venn diagram of putative venom proteins combining transcriptomic and proteomic analyses. Pv, *P. vindemmiae*; Dm, *D. melanogaster*; VG, venom apparatus (including venom reservoirs and associated glands); Vr, venom reservoir; Vg, venom gland; UVG, upregulated unigenes in VG compared to CA, Proteome: identified unigenes from proteome; SP, unigenes with a secretory signal peptide in their amino acid sequences; NSP, unigenes without a secretory signal peptide in their amino acid sequences. **(C)** Categories of the 64 putative *P. vindemmiae* venom proteins based on annotations in the Nr database.

*Pachycrepoideus vindemmiae* venom proteins (20 μg) were analyzed by 12 % SDS-PAGE. As shown in Figure 1A, venom proteins’ molecular weights ranged from 12 kDa to more than 180 kDa and 38 kDa venom proteins were particularly abundant. After quality inspection, 100 μg original venom proteins were directly digested with trypsin and analyzed by LC-MS/MS. Raw reads from one proteome were generated, and the identified peptide fragments were searched against the translated transcriptomic sequences of VG. Finally, 1,706 unigenes from the venom reservoirs got matches in transcriptome of VG with a strict filtration standard (FDR < 0.01) (Supplementary Table S2).

### Identification of Putative Venom Proteins Combining Transcriptomic and Proteomic Analyses

Venom proteins were identified under the assumption that they would show significantly higher expression in VG relative to CA (335) and were confirmed by proteome (1706) (Figure 1B). Based on these standards, 122 unigenes were identified. It is noticeable that a venom gene does not have to be higher expressed in VG, such as *PpS1V* in *P. puparum* (Yan et al., 2017). Further research should be undertaken to investigate low abundance venom components. However, we decided to focus further on this set of genes showing significantly higher expression in VG relative to other tissues and present in the venom reservoir proteome.

Venom proteins secreted by parasitoid wasp’ venom gland cells are expected to be secretory (Yan et al., 2016). Therefore, unigenes with initiation codons of the 122 candidates were computationally translated into peptides followed by a signal peptide prediction. The remaining unigenes without initiation codons were searched against the Nr database and their best matched sequences were retrieved from NCBI as references for a signal peptide prediction. Ultimately, 64 proteins were identified as putative venom proteins based on enhanced expression in VG, proteomic analysis, and a predicted signal peptide (Figure 1B). Besides, the expression fold-changes of 64 putative venom proteins ranged from 6.66 to infinity in VG relative to CA (Table 1), consistent with their likely venom functions. These candidates were subjected to further study.

**TABLE 1:** Venom proteins identified in *P. vindemmiae* by combined transcriptomic and proteomic analyses.

Gene ID	VG1	VG2	VG3	Carcass	Log_2_ (VG readcount/CA readcount)	NR Description (Blastp)		
	FPKM	FPKM	FPKM	FPKM		Accession number	*E*-value	Putative function
**Hydrolases**								
Cluster-8535.6348	19557	19579	97.08	11	11.081	XP_008214647.1	1.10E−43	PREDICTED: serine proteinase stubble isoform X2 [*Nasonia vitripennis*]
Cluster-8535.6313	836.53	957.24	220.24	0.64	11.06	XP_001599566.1	2.50E−54	PREDICTED: ribonuclease Oy-like [*Nasonia vitripennis*]
Cluster-8535.6305	153.65	162.55	10.17	2.18	6.7107	XP_001600074.1	1.80E−186	PREDICTED: serine protease easter [*Nasonia vitripennis*]
Cluster-8535.6218	363.86	363.47	48	0	Inf	XP_008214285.1	1.70E−66	PREDICTED: chymotrypsin-1-like [*Nasonia vitripennis*]
Cluster-8535.6181	353.98	395.1	186.31	0	Inf	NP_001166082.1	2.70E−32	Serine protease 64 precursor [*Nasonia vitripennis*]
Cluster-8535.6139	48.33	62.27	13.84	0	Inf	XP_001600770.1	1.00E−104	PREDICTED: venom acid phosphatase Acph-1-like [*Nasonia vitripennis*]
Cluster-8535.6129	709.9	861.32	129.65	0.07	14.111	XP_001600770.1	8.40E−126	PREDICTED: venom acid phosphatase Acph-1-like [*Nasonia vitripennis*]
Cluster-8535.6096	787.64	840.59	55.25	0	Inf	XP_001599566.1	1.20E−50	PREDICTED: ribonuclease Oy-like [*Nasonia vitripennis*]
Cluster-8535.6041	722.84	819.52	290.18	0.13	13.254	NP_001155016.1	4.90E−86	Serine protease homolog 29 precursor [*Nasonia vitripennis*]
Cluster-8535.6027	392.38	479.24	22.41	0.04	13.94	NP_001166082.1	1.30E−47	Serine protease 64 precursor [*Nasonia vitripennis*]
Cluster-8535.5971	476.58	559.55	322.5	0	Inf	XP_001606746.2	2.60E−51	PREDICTED: venom metalloproteinase 2-like [*Nasonia vitripennis*]
Cluster-8535.5960	252	261.04	32.79	0.17	11.122	XP_008216710.1	3.70E−170	PREDICTED: lipase 3-like [*Nasonia vitripennis*]
Cluster-8535.5821	203.77	241.77	103.02	0	Inf	NP_001166092.1	8.60E−07	Serine protease 87 precursor [*Nasonia vitripennis*]
Cluster-8535.25150	356.01	388	123.85	0	Inf	XP_003424313.1	8.10E−113	PREDICTED: LOW QUALITY PROTEIN: xaa-Pro aminopeptidase 1 [*Nasonia vitripennis*]
Cluster-8535.25095	135.52	168.77	38.94	0	Inf	XP_003424313.1	1.80E−111	PREDICTED: LOW QUALITY PROTEIN: xaa-Pro aminopeptidase 1 [*Nasonia vitripennis*]
Cluster-8535.25089	270.84	86.54	74.05	0	Inf	NP_001164348.1	4.10E−70	Serine protease precursor [*Nasonia vitripennis*]
Cluster-8535.25073	164.9	179.82	73.26	0.04	12.665	XP_008214271.1	2.10E−106	PREDICTED: chymotrypsin-2-like [*Nasonia vitripennis*]
Cluster-8535.25068	300.9	349.25	22.27	0.04	13.668	XP_003424313.1	1.70E−111	PREDICTED: LOW QUALITY PROTEIN: xaa-Pro aminopeptidase 1 [*Nasonia vitripennis*]
Cluster-8535.25024	301.44	311.46	98.65	0.5	9.9185	XP_012262898.1	4.70E−120	PREDICTED: neprilysin-like [*Athalia rosae*]
Cluster-8535.25017	4271.4	4371.7	1724.6	0	Inf	NP_001155017.1	2.10E−78	Serine protease 33 precursor [*Nasonia vitripennis*]
Cluster-8535.24980	5233.6	5127.5	704.54	0	Inf	NP_001155087.1	3.50E−102	Endonuclease-like venom protein precursor [*Nasonia vitripennis*]
Cluster-8535.24978	536.25	533.46	19.91	0.58	10.384	ACA60733.1	3.10E−169	Venom acid phosphatase [*Pteromalus puparum*]
**Oxidordeuctase**								
Cluster-8535.6260	556.41	462.2	177.86	3.49	7.8363	XP_001600327.1	7.60E−225	PREDICTED: glucose-6-phosphate 1-dehydrogenase [*Nasonia vitripennis*]
**Transferase**								
Cluster-8535.10302	1306.7	1291.7	47.55	0.01	16.952	XP_001607488.2	1.70E−261	PREDICTED: gamma-glutamyltranspeptidase 1 [*Nasonia vitripennis*]
**Protease inhibitor**								
Cluster-8535.6176	97.85	125.3	19.56	0.05	11.552	NP_001164350.1	2.30E−23	Kazal type serine protease inhibitor-like venom protein 2 precursor [*Nasonia vitripennis*]
**Recognition/binding proteins**								
Cluster-8535.6319	1048.3	1079.4	68.23	1.3	10.211	XP_001604854.1	5.50E−235	PREDICTED: low-density lipoprotein receptor-related protein 2-like [*Nasonia vitripennis*]
Cluster-8535.6269	118.07	99.09	62.88	0.14	10.33	XP_003425456.1	5.30E−108	PREDICTED: low-density lipoprotein receptor-related protein 2-like [*Nasonia vitripennis*]
Cluster-8535.6043	691.26	901.49	234.82	1.7	9.5069	NP_001164343.1	2.80E−25	Chitin binding protein-like venom protein precursor [*Nasonia vitripennis*]
Cluster-8535.25625	20.94	19.82	55.3	0	Inf	XP_003425456.1	5.60E−108	PREDICTED: low-density lipoprotein receptor-related protein 2-like [*Nasonia vitripennis*]
Cluster-8535.25152	37.77	34.53	52.24	0	Inf	XP_003425456.1	5.40E−108	PREDICTED: low-density lipoprotein receptor-related protein 2-like [*Nasonia vitripennis*]
Cluster-8535.25072	208.28	169.21	41.39	0	Inf	XP_003425456.1	3.50E−221	PREDICTED: low-density lipoprotein receptor-related protein 2-like [*Nasonia vitripennis*]
Cluster-8535.24989	856.24	894.02	42.53	1.12	9.955	XP_001604854.1	1.20E−35	PREDICTED: low-density lipoprotein receptor-related protein 2-like [*Nasonia vitripennis*]
**Others**								
Cluster-8535.6175	494.74	415.13	870.57	0.05	14.205	NP_001155022.1	1.80E−18	Cysteine-rich/TIL venom protein 2 precursor [*Nasonia vitripennis*]
Cluster-8535.6002	206.01	173.37	110.46	0.75	8.7008	XP_001604583.1	4.40E−37	PREDICTED: probable salivary secreted peptide [*Nasonia vitripennis*]
Cluster-8535.5294	188.51	143.89	210.12	0.14	11.104	XP_003708569.1	9.20E−08	PREDICTED: venom allergen 3-like [*Megachile rotundata*]
Cluster-8535.13847	138.85	179.77	66.68	0	Inf	XP_003423804.1	1.20E−42	PREDICTED: probable salivary secreted peptide [*Nasonia vitripennis*]
Cluster-8535.12135	67.17	30.36	14.72	0.36	7.6995	NP_001154978.1	2.80E−149	Major royal jelly protein-like 9 precursor [*Nasonia vitripennis*]
**Unknown proteins**								
Cluster-8535.6347	286.78	376.42	39.01	0	Inf	XP_011502473.1	3.50E−07	PREDICTED: uncharacterized protein LOC105365896 [*Ceratosolen solmsi marchali*]
Cluster-8535.6343	763.62	906.56	151.8	0.01	17.273	XP_008206401.1	5.70E−87	PREDICTED: uncharacterized protein LOC103316135 [*Nasonia vitripennis*]
Cluster-8535.6342	8523.7	8690.2	3580.7	0	Inf	XP_003426464.2	1.50E−23	PREDICTED: uncharacterized protein LOC100679170 [*Nasonia vitripennis*]
Cluster-8535.6295	7027	6416.4	2015.4	0	Inf	XP_008206613.1	1.80E−25	PREDICTED: venom protein H isoform X1 [*Nasonia vitripennis*]
Cluster-8535.6272	636.42	745.66	221.15	0	Inf	XP_003398548.1	3.60E−10	PREDICTED: uncharacterized protein LOC100649303 [*Bombus terrestris*]
Cluster-8535.6259	2842.6	2169.4	500.37	0.05	16.055	XP_003427828.1	4.90E−13	PREDICTED: uncharacterized protein LOC100678638 [*Nasonia vitripennis*]
Cluster-8535.6248	1980.5	2043.6	2440.1	0	Inf	XP_003424286.1	1.10E−17	PREDICTED: uncharacterized protein LOC100678044 [*Nasonia vitripennis*]
Cluster-8535.6242	301.5	302.67	157.3	0.02	15.009	XP_008206401.1	7.90E−83	PREDICTED: uncharacterized protein LOC103316135 [*Nasonia vitripennis*]
Cluster-8535.6206	915.67	1327.3	451.62	0.16	13.398	XP_001606832.1	1.30E−42	PREDICTED: uncharacterized protein LOC100123223 [*Nasonia vitripennis*]
Cluster-8535.6090	1490.1	1773.6	1235.4	0.38	12.858	NP_001155029.1	9.00E−19	Venom protein L precursor [*Nasonia vitripennis*]
Cluster-8535.6065	408.8	511.71	390.53	0	Inf	NP_001155028.1	1.30E−13	Venom protein K precursor [*Nasonia vitripennis*]
Cluster-8535.5978	194.01	237.28	19.94	0.07	12.229	XP_001601835.2	8.40E−206	PREDICTED: uncharacterized protein LOC100117668 [*Nasonia vitripennis*]
Cluster-8535.5949	251.98	157.94	283.57	0.16	11.384	NP_001164349.1	4.50E−69	Venom protein N precursor [*Nasonia vitripennis*]
Cluster-8535.5822	566.96	673.72	346.48	0	Inf	XP_008214317.1	1.20E−39	PREDICTED: uncharacterized protein LOC103317616 [*Nasonia vitripennis*]
Cluster-8535.25723	145.24	149.64	77.56	0.02	13.436	XP_001603409.1	5.60E−240	PREDICTED: uncharacterized protein LOC100119678 [*Nasonia vitripennis*]
Cluster-8535.25580	27.42	27.49	16.69	0.2	7.8349	NP_001155170.1	9.50E−44	Venom protein U precursor [*Nasonia vitripennis*]
Cluster-8535.25386	81.38	101.64	22.74	0.34	8.7077	XP_001601022.2	2.00E−250	PREDICTED: uncharacterized protein LOC100116563 [*Nasonia vitripennis*]
Cluster-8535.25243	38.36	37.63	10.68	0.06	9.947	XP_008217420.1	2.60E−14	PREDICTED: venom protein J isoform X1 [*Nasonia vitripennis*]
Cluster-8535.25157	41.59	38.61	10.18	0.36	7.4324	NP_001155170.1	1.90E−44	Venom protein U precursor [*Nasonia vitripennis*]
Cluster-8535.25146	216.01	280.26	82.17	0	Inf	XP_008210187.1	1.00E−50	PREDICTED: uncharacterized protein LOC103316723 [*Nasonia vitripennis*]
Cluster-8535.25086	695.81	768.91	450.62	0.03	15.253	NP_001155041.1	4.00E−14	Venom protein V precursor [*Nasonia vitripennis*]
Cluster-8535.25078	393.08	470.5	33.21	0.18	11.769	XP_008210187.1	3.80E−240	PREDICTED: uncharacterized protein LOC103316723 [*Nasonia vitripennis*]
Cluster-8535.24993	168.07	87.31	15.91	0.04	12.065	XP_001601177.1	1.50E−203	PREDICTED: uncharacterized protein LOC100116763 [*Nasonia vitripennis*]
Cluster-8535.17059	1212.9	1223.6	588.32	4.33	8.839	XP_003424551.1	1.80E−43	PREDICTED: uncharacterized protein LOC100680008 [*Nasonia vitripennis*]
Cluster-8535.16907	309.8	281.19	22.68	1.87	7.8547	XP_008210468.1	2.00E−303	PREDICTED: uncharacterized protein LOC100680448 isoform X3 [*Nasonia vitripennis*]
Cluster-8535.16090	270.75	290.36	182.01	0.38	10.333	XP_001604126.2	0.00E+00	PREDICTED: uncharacterized protein LOC100120484 [*Nasonia vitripennis*]
Cluster-8535.12555	124.88	120.27	64.48	0	Inf	XP_001604126.2	0.00E+00	PREDICTED: uncharacterized protein LOC100120484 [*Nasonia vitripennis*]

### Verification of Putative Venom Proteins by qPCR

**FIGURE 2:**
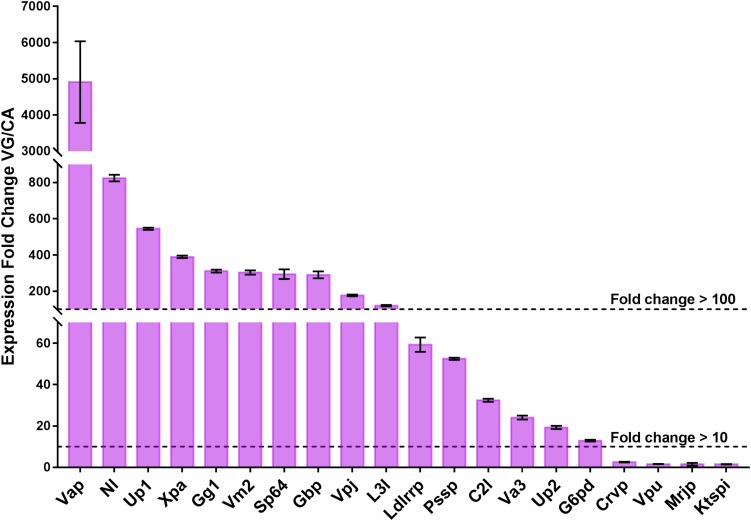
qPCR verification of the 20 selected putative venom proteins. The expression levels of 20 venom proteins in VG are normalized to their mean expression levels in CA, and shown as the mean ± standard deviation. The primers are listed in Supplementary Table S3. Gene full names and sequence accession numbers are provided as follows. Xpa, Cluster-8535.25068, xaa-Pro aminopeptidase; Up1, Cluster-8535.6242, uncharacterized protein LOC103316135; Va3, Cluster-8535.5294, venom allergen 3-like; Up2, Cluster-8535.16090, uncharacterized protein LOC100120484; C2l, Cluster-8535.25073, chymotrypsin-2-like; Gbp, Cluster-8535.6043, chitin binding protein-like venom protein precursor; Vm2, Cluster-8535.5971, venom metalloproteinase 2-like; Mrjp, Cluster-8535.12135, major royal jelly protein-like 9 precursor; Vap, Cluster-8535.24978, venom acid phosphatase; Ldlrrp, Cluster-8535.6269, low-density lipoprotein receptor-related protein 2-like; Crvp:, Cluster-8535.6175, cysteine-rich/TIL venom protein 2 precursor; L3l, Cluster-8535.5960, lipase 3-like; Nl, Cluster-8535.25024, neprilysin-like; Gg1, Cluster-8535.10302, gamma-glutamyltranspeptidase 1; Vpj, Cluster-8535.25243, venom protein J isoform X1; Ktspi, Cluster-8535.6176, Kazal type serine protease inhibitor-like venom protein 2 precursor; Pssp, Cluster-8535.6002, probable salivary secreted peptide; G6pd, Cluster-8535.6260, glucose-6-phosphate 1-dehydrogenase; Sp64, Cluster-8535.6027, serine protease 64 precursor; Vpu, Cluster-8535.25580, venom protein U precursor. The experiments were repeated 3 times.

Venom proteins can be more broadly expressed in the wasp VG, and highly specialized for their functions (Siebert et al., 2015; Martinson et al., 2017). To reveal the expression levels of 64 putative venom proteins in VG and to contrast with CA, we randomly selected 20 venom genes for qPCR verification. The expression fold-changes of 20 tested genes ranged from 1.50 to 4909.41, with a mean value of 418.53 and 10 of those (50%) showed greater than 100-fold increase in VG relative to CA, with 16 (80%) greater than 10-fold (Figure 2), indicating their specialization for venom functions. The remaining genes are more probably multifunctional (Martinson et al., 2017). This result also accorded with the data of high throughput RNA sequencing for quantifying the transcriptional levels of venom genes.

### Classification of *Pachycrepoideus vindemmiae* Putative Venom Proteins

According to the annotations of 64 putative venom proteins in the Nr database, 37 proteins could be assigned to functional categories (hereafter called “knowns”), while 27 could not (hereafter called “unknowns”) (Table 1). We noted that these functional categories were inferred by protein sequence similarities to annotated proteins from other organisms, or to recognized protein motifs. The functional categories of 37 “knowns” fell into hydrolases, oxidoreductases, transferases, protease inhibitors, recognition and binding proteins, and others (Figure 1C). Of the 37 categories, 22 hydrolases occupied the majority, and proteins commonly found in parasitoid venoms, such as serine proteases, metalloproteinase, acid phosphatases were included. This result is consistent with previous studies in pteromalid venom proteins, also demonstrated that hydrolases were the most abundant category (De Graaf et al., 2010; Yan et al., 2016). Furthermore, an oxidoreductase, a transferase, a protease inhibitor, five others, and seven venom proteins involved in the recognition and binding activities were also identified.

#### Hydrolases

**FIGURE 3:**
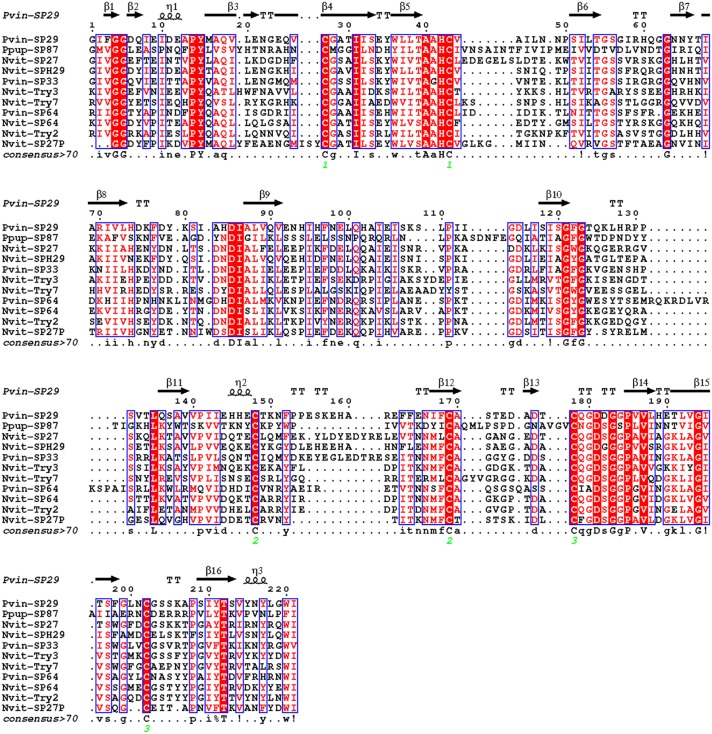
Multiple sequence alignment of “Tryp_SPc domain” in SPs and SPHs. Multiple alignment was conducted using Clustal Omega and visualized by ESPript 3.0. Protein full names and sequence accession numbers are provided as follows. Pvin-SP29, *P. vindemmiae* serine protease homolog 29 precursor (Cluster-8535.6041); Pvin-SP33, *P. vindemmiae* serine protease 33 precursor (Cluster-8535.25017); Pvin-SP64, *P. vindemmiae* serine protease 64 precursor (Cluster-8535.6181); Ppup-SP87, *P. puparum* serine protease 87 precursor (comp43143_c1) (Yan et al., 2016); Nvit-SP64, *N. vitripennis* serine protease 64 precursor (NP_001166082.1); Nvit-Try2, *N. vitripennis* trypsin-2-like (XP_016843706.1); Nvit-SP27, *N. vitripennis* serine protease 33 precursor (NP_001155017.1); Nvit-Try3, *N. vitripennis* trypsin-3 (XP_001603705.2); Nvit-SP27P, *N. vitripennis* serine protease 27 precursor (NP_001166077.1); Nvit-SPH29, *N. vitripennis* serine protease homolog 29 precursor (NP_001155016.1); Nvit-Try7, *N. vitripennis* trypsin-7 (XP_016838732.1). Pvin-VAP, *P. vindemmiae* venom acid phosphatase (Cluster-8535.24978); Pvin-VAPA, *P. vindemmiae* venom acid phosphatase Acph-1-like (Cluster-8535.6129); Ppup-VAP, *P. puparum* venom acid phosphatase (ACA60733.1); Nvit-VAPP, *N. vitripennis* venom acid phosphatase-like precursor (NP_001155147.1); Nvit-VAPA1, *N. vitripennis* venom acid phosphatase Acph-1-like (XP_001605452.1); Nvit-VAPA1.1, *N. vitripennis* venom acid phosphatase Acph-1-like (XP_001600770.1); Lhet-VAPA1.1, *L. heterotoma* venom acid phosphatase Acph-1-like1 (comp1442_c0_seq1); Lhet-VAPA1.2, *L. heterotoma* venom acid phosphatase Acph-1-like2 (comp2636_c0_seq1); Lbou-VAPA1, *L. boulardi* venom acid phosphatase Acph-1-like (comp9544_c0_seq1); Tpre-VAPA1.2, *Trichogramma pretiosum* venom acid phosphatase Acph-1-like isoform X1 (XP_014234174.2); Fari-VAPA, *Fopius arisanus* venom acid phosphatase Acph-1-like (XP_011310108.1); Tsar-VAP, *T. sarcophagae* venom acid phosphatase (OXU23470.1); Mpha-VAPA, *Monomorium pharaonis* venom acid phosphatase Acph-1-like (XP_012537166.1); Ccin-VAPA, *Cephus cinctus* venom acid phosphatase Acph-1 (XP_015589422.1); Sinv-VAP, *Solenopsis invicta* venom acid phosphatase (XP_025987662.1); Veme-VAP1, *Vollenhovia emeryi* venom acid phosphatase Acph-1-like isoform X1 (XP_011864393.1); Veme-VAP2, *V. emeryi* venom acid phosphatase Acph-1-like (XP_011872642.1); Aros-VAP, *A. rosae* venom acid phosphatase Acph-1-like (XP_012251812.1).

In this study, 10 serine proteases (SPs) and serine protease homologs (SPHs) were identified in *P. vindemmiae* venom proteins, including the typical chymotrypsin. The functions of SPs have been thoroughly investigated in *D. melanogaster*, one of the predominant hosts of *P. vindemmiae*, indicating that these SPs were involved in the activation of Toll immune pathway and prophenoloxidase (PPO) cascade reaction (Jang et al., 2008). In contrast, researches on parasitoid venom SPHs have been paid more attention. For instance, a serine proteinase homolog Vn50 of *C. rubecula* interfered with the proteolytic cascade and inhibited the melanization of host hemolymph (Asgari et al., 2003; Zhang et al., 2004a). Recently, combined genomic and transcriptomic approaches identified six SPs and two SPHs in *P. puparum* venom proteins, and some SPs showed higher expression levels after immune stimulation, implying that they might participate in antimicrobial immunity processes (Yang et al., 2017). As demonstrated in previous studies, the “Tryp_SPc domain” is crucial for SPs and SPHs (Jang et al., 2008; Veillard et al., 2016). According to this point, we aligned the “Tryp_SPc domain” of SPH (Cluster-8535.6041) and SPs (Cluster-8535.25017, Cluster-8535.6181) from *P. vindemmiae* with those from *N. vitripennis* and *P. puparum*, and uncovered many conservative residues between *P. vindemmiae* and its relatives (Figure 3). Thus, we infer that venom SPs and SPHs of *P. vindemmiae* suppresses the host humoral immunity in similar manners to other pteromalids.

The ribonuclease Oy-like (Cluster-8535.6096) and endonuclease-like venom proteins (Cluster-8535.24980) of *P. vindemmiae* display higher identities with *N. vitripennis* (Blastp, 60% and 43%, respectively). A possible speculation for the presence of nucleases in *P. vindemmiae* venoms was that they cleave RNA of the host to confront its defensive responses (Trummal et al., 2016).

**FIGURE 4:**
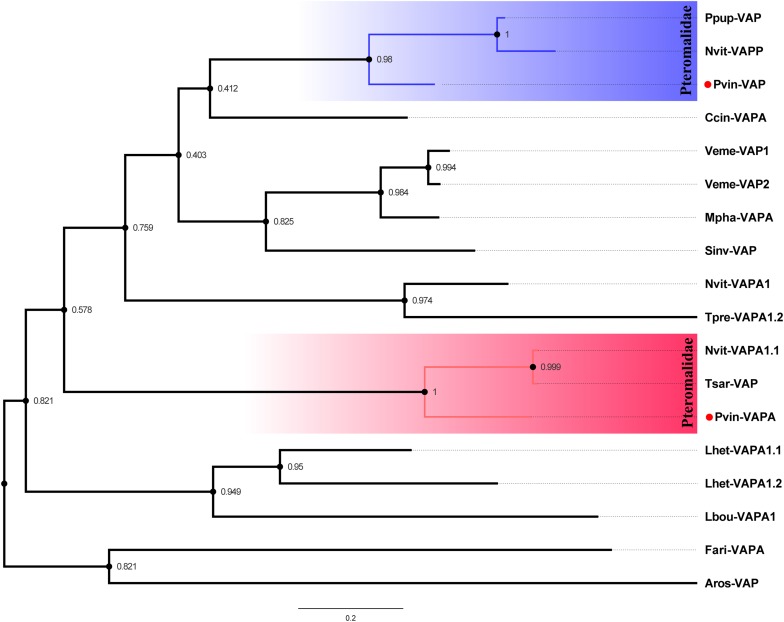
Phylogenetic reconstruction based on “His_Phos_2 domain” of venom acid phosphatases. The phylogenetic tree was constructed by maximum likelihood method using the program Mega 6. 48 different amino acid substitution models were tested and the “LG+G+I” was considered to be the best model. The bootstrap values are presented on the nodes. Both venom proteins and non-venom orthologs were used for phylogenetic analysis. Full names of abbreviations are listed as follows. Pvin-VAP, *P. vindemmiae* venom acid phosphatase (Cluster-8535.24978); Pvin-VAPA, *P. vindemmiae* venom acid phosphatase Acph-1-like (Cluster-8535.6129); Ppup-VAP, *P. puparum* venom acid phosphatase (ACA60733.1); Nvit-VAPP, *N. vitripennis* venom acid phosphatase-like precursor (NP_001155147.1); Nvit-VAPA1.1, *N. vitripennis* venom acid phosphatase Acph-1-like (XP_001600770.1); Lhet-VAPA1.1, *L. heterotoma* venom acid phosphatase Acph-1-like1 (comp1442_c0_seq1); Lhet-VAPA1.2, *L. heterotoma* venom acid phosphatase Acph-1-like2 (comp2636_c0_seq1); Lbou-VAPA1, *L. boulardi* venom acid phosphatase Acph-1-like (comp9544_c0_seq1); Tpre-VAPA1.2, *Trichogramma pretiosum* venom acid phosphatase Acph-1-like isoform X1 (XP_014234174.2); Fari-VAPA, *Fopius arisanus* venom acid phosphatase Acph-1-like (XP_011310108.1); Tsar-VAP, *T. sarcophagae* venom acid phosphatase (OXU23470.1); Mpha-VAPA, *Monomorium pharaonis* venom acid phosphatase Acph-1-like (XP_012537166.1); Ccin-VAPA, *Cephus cinctus* venom acid phosphatase Acph-1 (XP_015589422.1); Sinv-VAP, *Solenopsis invicta* venom acid phosphatase (XP_025987662.1); Veme-VAP1, *Vollenhovia emeryi* venom acid phosphatase Acph-1-like isoform X1 (XP_011864393.1); Veme-VAP2, *V. emeryi* venom acid phosphatase Acph-1-like (XP_011872642.1); Aros-VAP, *A. rosae* venom acid phosphatase Acph-1-like (XP_012251812.1).

Three venom acid phosphatases (Cluster-8535.6129, Cluster-8535.6139, and Cluster-8535.24978) packed with a conserved “His_Phos_2 domain” were originally identified in *P. vindemmiae* venom proteins. As commonly known venom components in hymenopteran parasitoids, venom acid phosphatases also exist in *P. puparum* (Zhu et al., 2008), *L. heterotoma* (Heavner et al., 2013), *L. boulardi* (Colinet et al., 2013), *N. vitripennis* (Danneels et al., 2010), *Pimpla hypochondriaca* (Dani et al., 2005), and *Hyposoter didymator* (Doremus et al., 2013). Based on the phylogenetic analysis of “His_Phos_2 domain,” we found that *P. vindemmiae* venom acid phosphatase (Cluster-8535.24978) and venom acid phosphatase Acph-1-like (Cluster-8535.6129) evolved into two branches (Figure 4), both clustering together with other pteromalids. Although studies noted the importance of venom acid phosphatases in parasitoids (Zhu et al., 2008), there were few exhaustive studies yet. We propose that the venom acid phosphatases of *P. vindemmiae* may play a special role in affecting the host’s physiology and acquiring nutrients from the host hemolymph (Xia et al., 2000; Zhu et al., 2008).

A venom protein of *P. vindemmiae* was annotated as metalloproteinase (Cluster-8535.5971). Metalloproteinases also present in *N. vitripennis* (De Graaf et al., 2010), *Chelonus inanitus* (Vincent et al., 2010), *L. boulardi* and *L. heterotoma* (Goecks et al., 2013), *H. didymator* (Doremus et al., 2013), *Microctonus hyperodae* (Crawford et al., 2008), *Microplitis demolitor* (Burke and Strand, 2014; Lin et al., 2018), and *Eulophus pennicornis* (Price et al., 2009). It was shown that recombinant venom protein metalloproteinase from *E. pennicornis* resulted in partial host mortality during molt and a delay in growth and development of the host (Price et al., 2009). Moreover, a compelling study suggested that the metalloproteinase from *M. mediator* venom reservoir interfered with host immune signaling cascades by binding to host nuclear factor kappa B (Lin et al., 2018). These studies will provide clues for functional characterization of metalloproteinase in *P. vindemmiae* venom proteins.

A lipase (Cluster-8535.5960) exists in *P. vindemmiae* venom reservoirs. Previous studies showed that *N. vitripennis* venom proteins induced alteration in lipid metabolism and arrested larval development of the host, implying that the lipase is an indispensable part of venom proteins (Rivers and Denlinger, 1995; Mrinalini et al., 2014). There is an abundant room for determining the specific mechanism of lipase from *P. vindemmiae* venom proteins in regulating host lipid metabolism.

In this study, three aminopeptidases (Cluster-8535.25150, Cluster-8535.25095, and Cluster-8535.25068) were originally identified in *P. vindemmiae* venom proteins. In previous documented literatures, peptidases are crucial components in endoparasitoid venom proteins (Yan et al., 2016; Teng et al., 2017). It is inferred that aminopeptidases of *P. vindemmiae* are involved in the hydrolysis of host peptides, and further provide essential amino acids required for growth and development of offspring.

We identified a neprilysin in *P. vindemmiae* venom proteins (Cluster-8535.25024). Neprilysins have been characterized in many fly parasitoids, such as *Psyttalia lounsburyi*, *Psyttalia concolor* (Hubert et al., 2016), *L. boulardi* (Goecks et al., 2013), and *Ganaspis* sp. 1 (Mortimer et al., 2013). There is a great possibility that venom protein neprilysin modulates the host’s immune responses. More investigations about their roles in parasitism are imperatively needed.

#### Oxidoreductase

An oxidoreductase of *P. vindemmiae* venom proteins was annotated as glucose-6-phosphate 1-dehydrogenase (Cluster-8535.6260). Glucose dehydrogenases also exist in venom reservoirs of *P. puparum* (Yan et al., 2016), *N. vitripennis* (De Graaf et al., 2010), *L. boulardi* and *L. heterotoma* (Goecks et al., 2013). We assume that it participates in the carbohydrate catabolism of the host and provides nutrition for the development of parasitoid offspring.

#### Transferase

Here we originally identified a gamma-glutamyltranspeptidase (Cluster-8535.10302) in *P. vindemmiae* venom proteins. Venom protein gamma-glutamyltranspeptidase was exhaustively described in *Aphidius ervi*, inducing cell apoptosis of the host ovariole by altering GSH metabolism and oxidative stress (Falabella et al., 2007). Whether the venom gamma-glutamyltranspeptidase of *P. vindemmiae* performs functions similar to that of *A. ervi* needs to be investigated.

#### Protease Inhibitor

**FIGURE 5:**
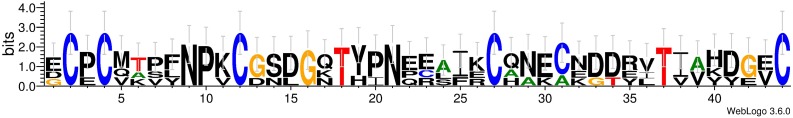
Motif-based sequence analysis of “Kazal domain” in pteromalids. The sequence logo was generated using WebLogo based on “Kazal domain” of *P. vindemmiae* (Cluster-8535.6176), *N. vitripennis* (NP_001164350.1) and *P. puparum* (comp22195_c0) (Yan et al., 2016).

A Kazal-type serine protease inhibitor (Cluster-8535.6176) was characterized in *P. vindemmiae* venom proteins. Similar with other parasitoids, the Kazal-type serine protease inhibitor of *P. vindemmiae* is packed by a conservative “Kazal domain.” The motif prediction based on “Kazal domain” revealed several conservative amino acid residues between *P. vindemmiae*, *N. vitripennis* and *P. puparum* (Figure 5). Previous study showed that Kazal-type serine protease inhibitors from *N. vitripennis* venom proteins inhibited the PPO activation of host hemolymph (Qian et al., 2015), strongly supporting the speculation that the Kazal-type serine protease inhibitor from *P. vindemmiae* venom proteins may suppress the host humoral immunity, especially in melanization.

#### Recognition and Binding Proteins

Low density lipoprotein receptors were extensively identified in parasitoids, including *P. vindemmiae* (Cluster-8535.6319, Cluster-8535.6269, Cluster-8535.25625, Cluster-8535.25152, Cluster-8535.25072 and Cluster-8535.24989), *P. puparum*, and *N. vitripennis* (De Graaf et al., 2010; Yan et al., 2016). It was first reported that an insect homolog of low density lipoprotein receptor mediated the endocytosis of high density lipophorin in the circulatory compartment (Dantuma et al., 1999). We infer that the low density lipoprotein receptors of *P. vindemmiae* venom proteins participate in the internalization of high density lipophorin (Danneels et al., 2010).

Other than low density lipoprotein receptors, a venom protein annotated as chitin binding protein-like (Cluster-8535.6043) was also identified in *P. vindemmiae*. It was reported that chitin binding proteins present in the venom proteins of *N. vitripennis* and *P. puparum* (De Graaf et al., 2010; Zhu et al., 2015). Based on the previous investigation, we suspect that the chitin binding venom protein of *P. vindemmiae* selectively binds chitin, and likely facilitates wound healing of the host exoskeleton (Zhu et al., 2015).

#### Others

Venom allergens are common components in stinging insects, such as bees, fire ants and vespids. In *P. vindemmiae*, the venom allergen (Cluster-8535.5294) showing 25% identity (Blastp, *e*-value = 9e^–08^) with *M*. *demolitor* venom allergen 5 possibly leads to the anaphylaxis of the host.

In hymenopteran insects, major royal jelly proteins are necessary for social behavior. They were identified in *P. vindemmiae* (Cluster-8535.12135) and *P. puparum* (Yan et al., 2016) venom proteins. In honeybee, the royal jelly protein regulated the larval development, induced queen differentiation (Peiren et al., 2005, 2008), and embodied the novel nutritious function as major components of royal jelly (Klaudiny, 2007). Hereby, we infer that the major royal jelly venom protein of *P. vindemmiae* is related to the storage of nutrients.

Alongside the above-mentioned proteins, a venom protein was annotated as cysteine-rich peptide (Cluster-8535.6175) in *P. vindemmiae*, and highly expressed in VG. In accordance with the previous study in *Nasonia* venom proteins (De Graaf et al., 2010), the cysteine-rich peptide of *P. vindemmiae* contains six conservative cysteine residues forming three disulfide bridges. Its function in parasitism is an important issue for future research. Besides, one unanticipated observation was the category of two salivary secreted peptides (Cluster-8535.6002, Cluster-8535.13847). It is speculated that they function in hydrolyzing the host nutrients.

#### Unknown Proteins

A total of 27 *P. vindemmiae* venom proteins were categorized into “unknowns” based upon the absence of recognized protein motifs. In contrast to our current study, 23 and 17 “unknowns” were originally identified in venom proteins of other pteromalids, *N. vitripennis* and *P. puparum*, respectively (De Graaf et al., 2010; Yan et al., 2016). Comparing the 27 “unknowns” venom expression of *P. vindemmiae* to that of the 37 “knowns” showed a similar expression pattern, ranging from 7.43 to 17.27-fold higher in VG than that in CA (mean value of 11.76) among “unknowns” compared to 6.71-16.95-fold higher (mean value of 11.18) among “knowns.”

### Comparative Analysis of Parasitoid Venom Proteins

**FIGURE 6:**
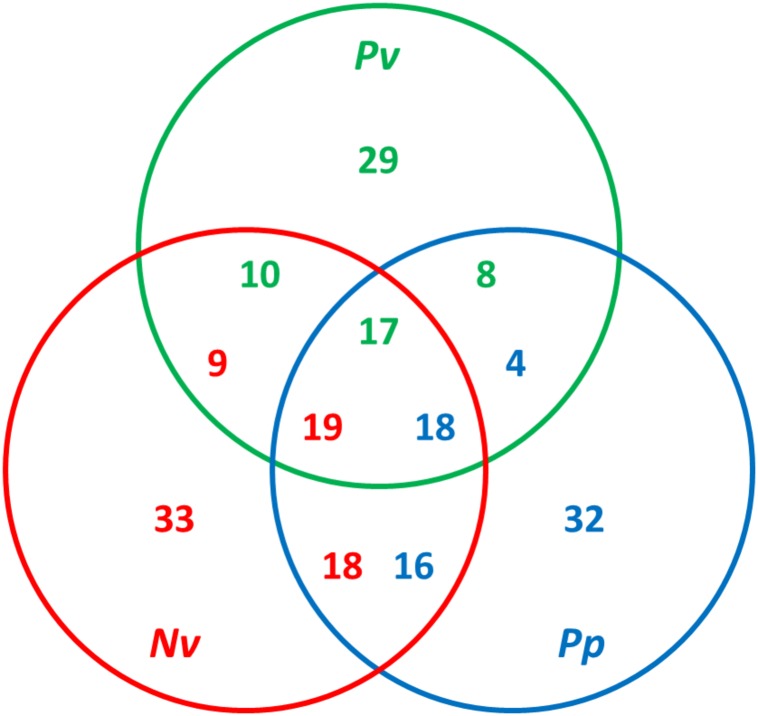
Comparative analysis of venom proteins in pteromalids. The orthologs were identified using OrthoMCL by all-against-all blastp with a *p*-value cut-off of 1e^–5^. The green numbers indicate hits from *P. vindemmiae* venoms, the red numbers indicate hits from *N. vitripennis* venoms, and the blue numbers indicate hits from *P. puparum* venoms. The numbers of orthologs can be different in different venom sets. *Pv*, venom proteins of *P. vindemmiae*; *Nv*, venom proteins of *N. vitripennis*; *Pp*, venom proteins of *P. puparum*.

Previous studies have found both considerable diversity of venom proteins between species and also evidence of functional redundancy within species (Goecks et al., 2013; Yan et al., 2016; Martinson et al., 2017). Hereby, a comparative analysis was conducted using OrthoMCL by all-against-all blastp on the basis of a set of venom protein sequences from *P. vindemmiae* and other pteromalids (*N. vitripennis* and *P. puparum*) (Doerks et al., 2002). Putative orthologs were identified by reciprocal best matches. Based on this criterion, thirty-five venom proteins of *P. vindemmiae* were assigned to orthologous groups, while the remaining 29 were not. Figure 6 shows a Venn diagram of orthologous relationships between *P. vindemmiae* venom proteins and those of *N. vitripennis*, *P. puparum*. Among the 35 orthologs in *P. vindemmiae*, 27 have orthologous relationships with *N. vitripennis* venom proteins and 25 with venoms of *P. puparum* (Figure 6). Besides, 23 “knowns” and 12 “unknowns” were included in the 35 categorized orthologs. As can be seen, the proportion of “unknown” orthologs in total “unknown” venom proteins (12 of 27) was not different from that of “knowns” (23 of 37) (*p* = 0.52, Fisher Exact Test). Of the 23 “known” orthologs, 16 categories have orthologous relationships with *N. vitripennis* venom proteins and 18 with *P. puparum* venoms. These orthologs represent a set of conserved venom proteins in pteromalids, possibly contributing to their adaptation to parasitism. In contrast, of the 12 “unknown” orthologs, 11 have orthologous relationships with *N. vitripennis* venom proteins and 7 with *P. puparum* venoms, representing a set of unique venom proteins in pteromalids. Many of these venoms were highly expressed in VG. For instance, the expression level of venom protein J in VG was 176.73-fold higher than that in CA (Figure 2). The observations that these “unknowns” are orthologous with the related pteromalid species, do not have clear homologies to other parasitoid venom proteins, and have higher VG expression levels suggest their specialized venom functions.

**FIGURE 7:**
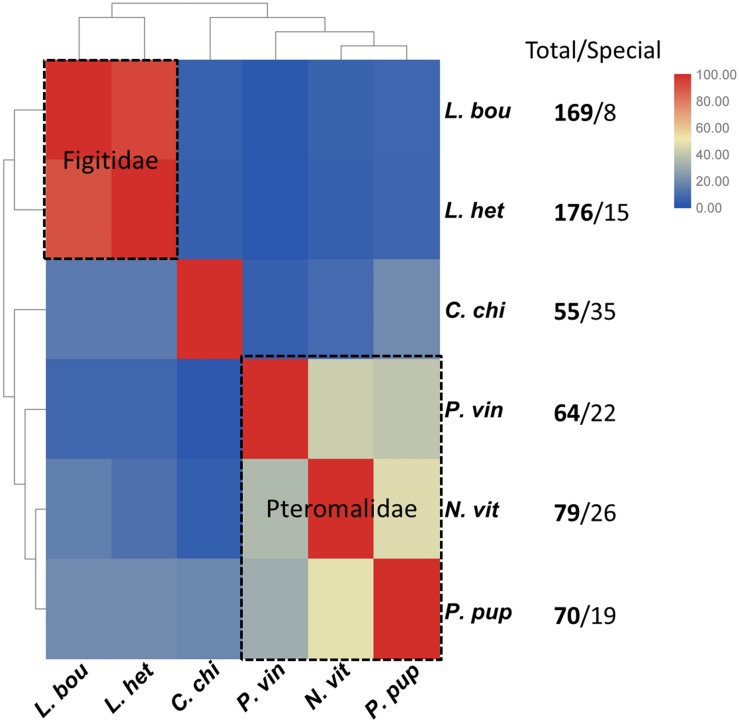
Cluster analysis of the orthologs in six parasitoids. The heatmap was plotted using TBtools v0.6669 based on the proportion of orthologs in total venom proteins (Chen et al., 2018). Red indicates larger proportion of the orthologs and blue indicates the smaller. Each grid shows the proportion of orthologous venom proteins in total venom proteins of the horizontal axis labeled species by reciprocal best matches with the venom proteins of vertical axis labeled species. *C. chi*, *C. chilonis*; *L. bou*, *L. boulardi*; *L. het*, *L. heterotoma*; *N. vit*, *N. vitripennis*; *P. pup*, *P. puparum*; *P. vin*, *P. vindemmiae*.

Additionally, we compared the venom proteins of *P. vindemminae* with those from other parasitoids (*L. boulardi*, *L. heterotoma*, and *C. chilonus*) encompassing two families, Figitidae and Braconidae. The sequences of venom proteins were obtained from previous literatures (Goecks et al., 2013; Teng et al., 2017), using species containing 50 or more venom proteins. Venom proteins of each species were assigned to orthologs according to the above classification criterion (Doerks et al., 2002). Results were displayed in clustered heatmap based on the proportion of orthologs in total venom proteins. As expected, closely related species shared a greater proportion of orthologs, even though they have more abundant venom proteins (e.g., *L. boulardi* and *L. heterotoma*). Two representative groups clustered together, one representing Pteromalidae and another representing Figitidae (Figure 7). Five *P. vindemminae* venom proteins (venom allergen: Cluster-8535.5294, serine protease: Cluster-8535.6305, venom acid phosphatase: Cluster-8535.24978, lipase: Cluster-8535.5960, and neprilysin: Cluster-8535.25024) in this set have orthologous relationships with venoms of Figitidae representatives, and two orthologs were identified in *P. vindemminae* venoms (“unknowns”: Cluster-8535.25078 and Cluster-8535.25723) by reciprocal best matches with venom proteins of the single representative of Braconidae. The large differences among this set of venoms may be due to their specific adaptations during the co-evolution between parasitic wasps and host species, and are consistent with the observed rapid turnover even in closely related species’ venom proteins (Goecks et al., 2013; Martinson et al., 2017).

### Phylogenetic Analysis of *Pachycrepoideus vindemmiae*

**FIGURE 8:**
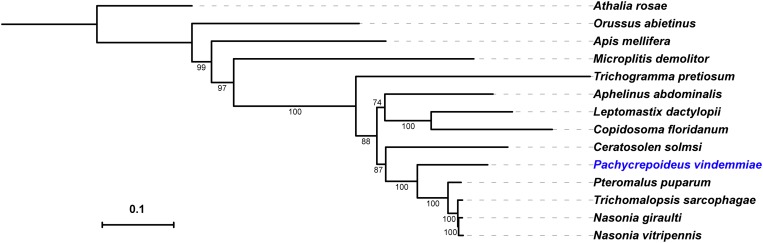
Phylogeny of *P. vindemmiae*. A phylogeny was generated based on a set of 107 proteins, and visualized using iTOL v3 (Letunic and Bork, 2016). Indicated are members of the Pternomalidae and the chalcidoid superfamily. Proteins sequences from *P. vindemmiae* (this study), *P. puparum* (GECT00000000.1), *Aphelinus abdominalis* (GBTK00000000.1), and *Leptomastix dactylopii* (GBNE00000000.1) transcriptomes were assigned to these 107 proteins groups. Bootstrap values are shown at each node. *P. vindemmiae* is labeled in blue.

To better understand the evolutionary position of *P. vindemmiae*, a phylogenetic tree was constructed based on a data set of 107 proteins across a larger set of single-copy orthologs described in Lindsey et al. (best-reciprocal hits with *p*-value cut-off < 1e^–5^) (Lindsey et al., 2018). What stood out in the Figure 8 was that *P. vindemmiae* clustered together with other pteromalids, namely *P. puparum, N. vitripennis*, *Trichomalopsis sarcophagae*, and *Nasonia giraulti*, suggesting that they have evolved from the same ancestor. The majority of pteromalids are specialists, such as *P. puparum*, *N. vitripennis* and *N. giraulti*. Our observation that *P. vindemmiae* has a relatively close evolutionary relationship with these specialists provides a wonderful model for comparing the phylogeny between specialist and generalist, particularly in the variations of parasitic behavior. In the closely related species, the generalist *L. boulardi* and specialist *L. heterotoma*, comparison of their venom proteins profiles revealed several different venom compositions (Poirié et al., 2014), and may thus reflect long-term adaptations to their hosts (Goecks et al., 2013). Our examination on orthologous venom proteins also provides clues about the different parasitic behavior between *P. vindemmiae* and other specialists in pteromalids. This finding also provides corroborative evidence that the venom components of pteromalids are somewhat similar, and lays a foundation for functional research on venom proteins of *P. vindemmiae*.

As a generalist in dampening oscillations of Dipteran Cyclorrhapha, *P. vindemmiae* possesses vast potentials on host-killing capacity. Several practical cases have addressed its high efficacy at reducing flies’ populations, one of which was that *P. vindemmiae* decreased the fitness of the host *Bactrocera oleae* (Hoelmer et al., 2011). A follow-up study also underlined that *P. vindemmiae* has been attracting considerable interest in control of *D. suzukii*, a destructive pest in American and European orchard (Bezerra Da Silva et al., 2019). More remarkably, the investigation into its ability to control *D. suzukii* populations showed an efficient reduce in host numbers by 32% (Bonneau et al., 2019). Our current study about the venom protein repertoire of ectoparasitoid *P. vindemmiae* will vastly propel its possible utilization as a biological agent to the control of flies. Taken as a whole, these explorative works expand our knowledge that *P. vindemmiae* has a tremendous biocontrol potential against flies.

## Conclusion

**FIGURE 9:**
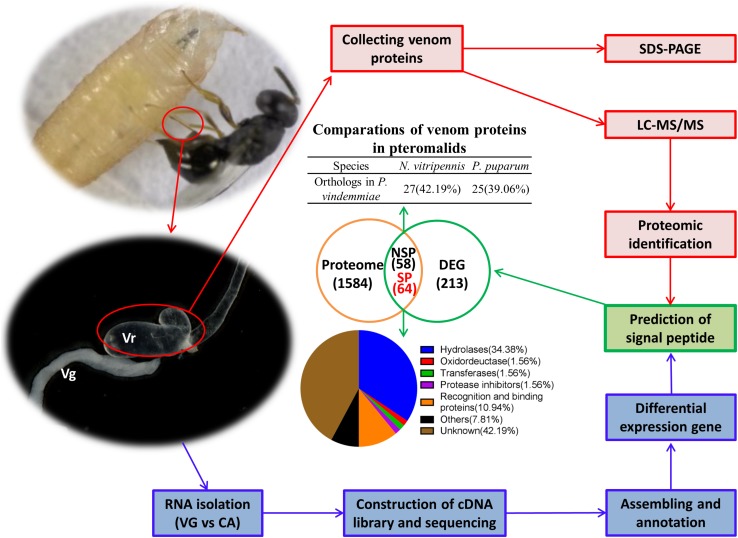
Schematic representation of the identified venom proteins in *P. vindemmiae* by combined transcriptomic and proteomic analyses.

In this study, we described the venom protein repertoire of *P. vindemmiae* (Figure 9), an ectoparasitoid that utilizes pupae of *Drosophila* species as a host. Both transcriptomic and proteomic analyses were used to identify 64 putative venom proteins in *P. vindemmiae*. Our striking observation revealed 35 core orthologous venom proteins from *P. vindemmiae* by best-reciprocal matches with other pteromalids. More distant relationships detected that five venom proteins of *P. vindemmiae* have orthologous relationships with venoms of Figitidae parasitoids and two with those of a Braconidae representative. In addition, twenty-two venoms unique to *P. vindemmiae* were detected, consistent with observations of rapid turnover and evolution of new parasitoid venom proteins. Our findings will deepen the understanding of the phylogeny as well as inner- and inter-taxon variations of venom proteins.

## Data Availability Statement

The datasets generated for this study can be found in the Venom apparatus and carcasses transcriptome of *Pachycrepoideus vindemmiae* (https://www.ncbi.nlm.nih.gov/sra/PRJNA573955), the mass spectrometry proteomic data of *Pachycrepoideus vindemmiae* were deposited on the ProteomeXchange Consortium with the dataset identifier PXD015627 (Ma et al., 2019).

## Ethics Statement

We declare that appropriate ethical approval and licenses were obtained during our research.

## Author Contributions

LY, YY, and M-ML performed the experiments. LY, Z-CY, L-MQ, and FW analyzed the data. LY, QF, JW, and G-YY designed the experiments. LY, JW, and G-YY wrote the manuscript. All authors gave final approval for publication.

## Conflict of Interest

The authors declare that the research was conducted in the absence of any commercial or financial relationships that could be construed as a potential conflict of interest.

## Footnotes

1 http://arthropods.eugenes.org/genes2/nasonia/genes/

## Supplementary Material

FIGURE S1 GO functional categories of the 335 UVG. The 335 UVG were annotated on the GO consortium database using the GOseq R packages with e-value < 1e^-6^.

FIGURE S2 Top 20 KEGG functional categories of the 335 UVG. KEGG annotations were implemented by KOBAS software with e-value < 1e^-10^.

TABLE S1 335 upregulated unigenes in VG compared to CA.

TABLE S2 1706 unigenes identified by proteomic approach.

TABLE S3 Primers used for qPCR analysis of 20 venom genes.
