# Supplementary figures and images for: Identification and Comparative Analysis of Venom Proteins in a Pupal Ectoparasitoid, Pachycrepoideus vindemmiae
Source: Front Physiol. 2020 Jan 24;11:9. doi: 10.3389/fphys.2020.00009 (PMC6993573; doi:10.3389/fphys.2020.00009)

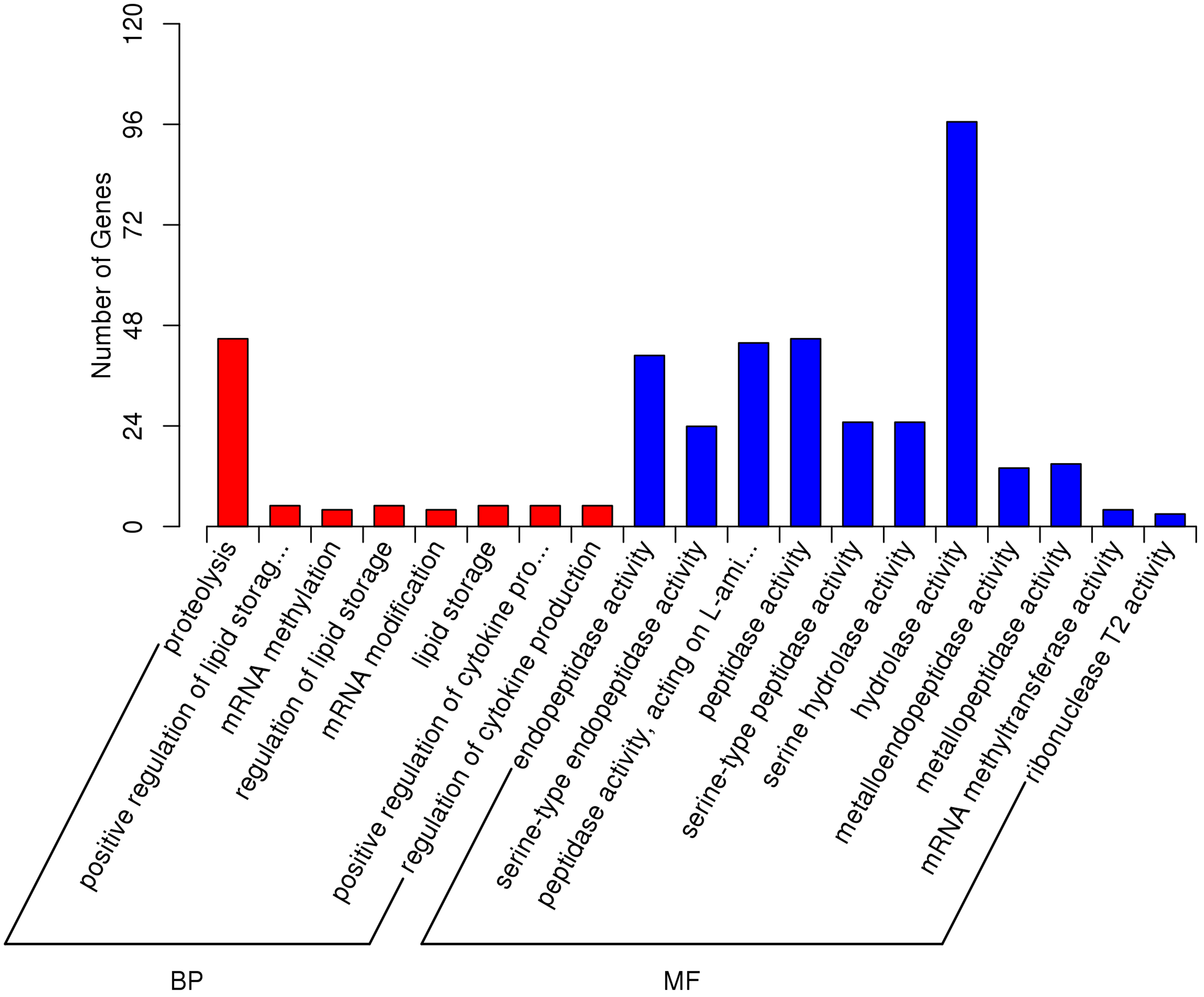

Supplement: FIGURE S1 — GO functional categories of the 335 UVG. The 335 UVG were annotated on the GO consortium database using the GOseq R packages with e-value < 1e-6. [file Image_1.TIF]

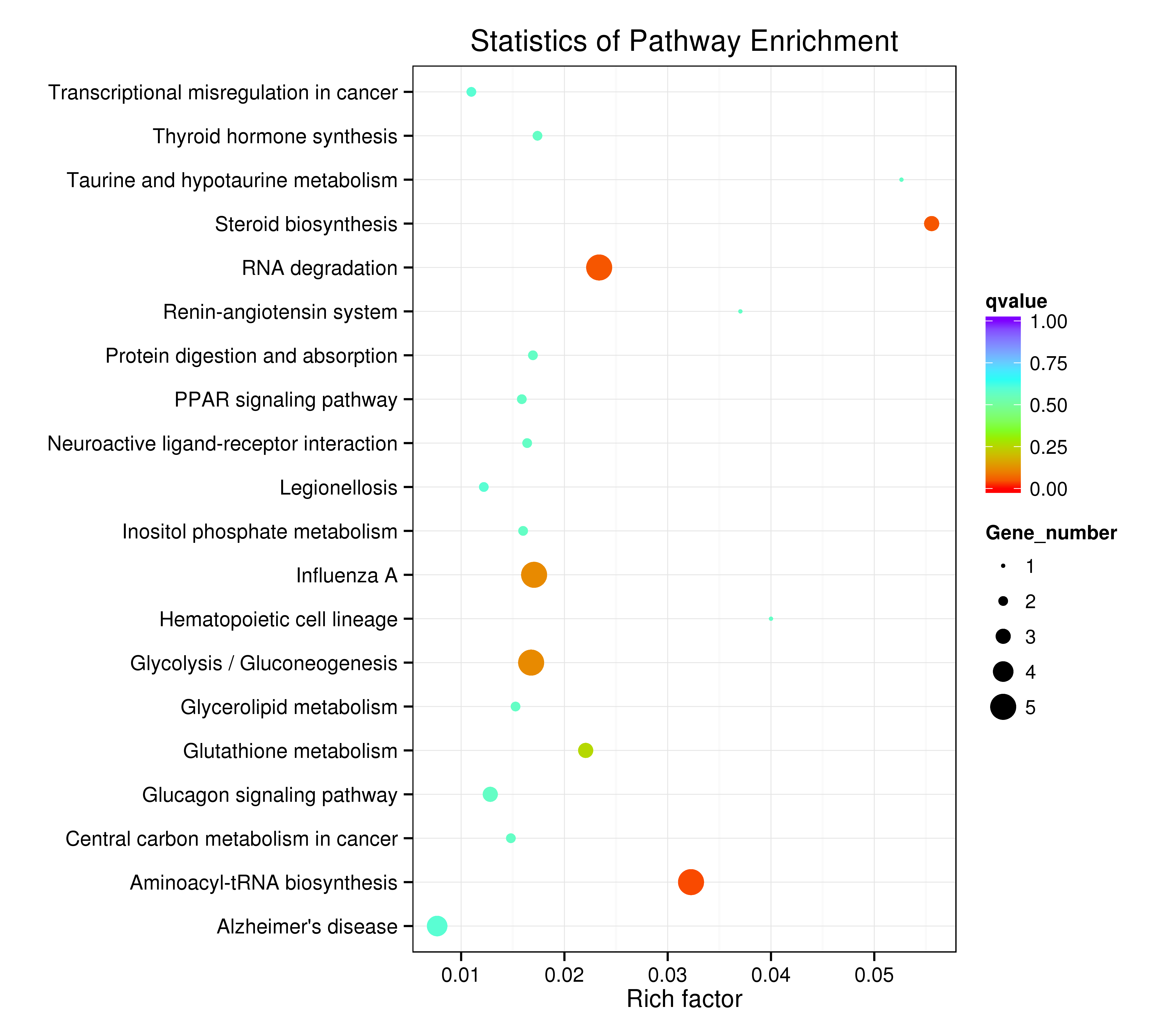

Supplement: FIGURE S2 — Top 20 KEGG functional categories of the 335 UVG. KEGG annotations were implemented by KOBAS software with e-value < 1e-10. [file Image_2.TIF]
